# A biodegradable chipless sensor for wireless subsoil health monitoring

**DOI:** 10.1038/s41598-022-12162-z

**Published:** 2022-05-14

**Authors:** Sarath Gopalakrishnan, Jose Waimin, Amin Zareei, Sotoudeh Sedaghat, Nithin Raghunathan, Ali Shakouri, Rahim Rahimi

**Affiliations:** 1 School of Electrical and Computer Engineering, Purdue University, West Lafayette, IN 47907 USA; 2 School of Materials Engineering, Purdue University, West Lafayette, IN 47907 USA; 3 Birck Nanotechnology Center, Purdue University, West Lafayette, IN 47907 USA

**Keywords:** Electrical and electronic engineering, Polymers, Sustainability

## Abstract

Precision Agriculture (PA) is an integral component of the contemporary agricultural revolution that focuses on enhancing food productivity in proportion to the increasing global population while minimizing resource waste. While the recent advancements in PA, such as the integration of IoT (Internet of Things) sensors, have significantly improved the surveillance of field conditions to achieve high yields, the presence of batteries and electronic chips makes them expensive and non-biodegradable. To address these limitations, for the first time, we have developed a fully Degradable Intelligent Radio Transmitting Sensor (DIRTS) that allows remote sensing of subsoil volumetric water using drone-assisted wireless monitoring. The device consists of a simple miniaturized resonating antenna encapsulated in a biodegradable polymer material such that the resonant frequency of the device is dependent on the dielectric properties of the soil surrounding the encapsulated structure. The simple structure of DIRTS enables scalable additive manufacturing processes using cost-effective, biodegradable materials to fabricate them in a miniaturized size, thereby facilitating their automated distribution in the soil. As a proof-of-concept, we present the use of DIRTS in lab and field conditions where the sensors demonstrate the capability to detect volumetric water content within the range of 3.7–23.5% with a minimum sensitivity of 9.07 MHz/%. Remote sensing of DIRTS can be achieved from an elevation of 40 cm using drones to provide comparable performance to lab measurements. A systematic biodegradation study reveals that DIRTS can provide stable readings within the expected duration of 1 year with less than 4% change in sensitivity before signs of degradation. DIRTS provides a new steppingstone toward advancing precision agriculture while minimizing the environmental footprint.

## Introduction

The first and continuing challenge facing global agriculture is to produce enough food that can meet the fast-growing population around the world. It is estimated that by 2050 the population will increase by 1.2 billion requiring a 90% increase in food demand to meet this need^1^. While expanding agricultural facilities is vital to meet this projected requirement, the environmental challenges posed by the mismanagement of agricultural resources, such as water^2^, arable land^3^, and fertilizers^4^, are enormous. Among the various agricultural reserves, water is one of the quintessential natural resources required for sustainable agriculture. Agriculture is the biggest consumer of water supplies with 70% of the freshwater on earth being used for crop cultivation^5^. However, poor management of water resources leads to irregular irrigation practices which give rise to serious environmental problems^6^. Over-irrigation leads to salinization^7^, alkalization^8^ and waterlogging^9^ of arable lands, and water pollution due to nitrogen leaching^10^, whereas under-irrigation leads to high vegetation dryness stress and poor crop yield^11,12^. The parameter that is critical in estimating the efficiency of irrigation is the volumetric water content (VWC) at the root zone of the crops. Studies have shown that optimizing VWC in the soil provides the best crop yield validated through a strong correlation between VWC and crop yield^13^.

In addition to irrigation efficiency and crop yield, VWC is a key indicator of microbial activity and plant health in the soil^14^. VWC has been identified as a significant biomarker of soil microorganisms that are responsible for the decomposition of organic matter^15^, nitrogen fixation^16^, and solubilization of phosphorus^17^. Since VWC is an important factor in the hydrological, biochemical, and economic aspects of farming, in-situ monitoring of the VWC of the soil is essential to improve the irrigation efficiency, crop yield, soil health, and subsequently achieve maximum food productivity. However, most agricultural fields often have high spatial variability in the soil moisture due to fluctuating topographical terrain attributes, such as litter decomposition, vegetation composition, and soil management practices^18^. This high heterogeneity of the agricultural land caused by the spatial variability of the soil is a major hurdle in achieving efficient resource allocation throughout agricultural fields.

Precision Agriculture (PA) can address the need for efficient resource allocation by creating a soil map of the entire field to monitor and distribute resources judiciously^19^. Several techniques have been adopted as part of PA to monitor soil conditions^20^. Among different approaches, wireless and remote sensing technologies are the most preferred as they provide significant practical value in large-scale agricultural fields. The state-of-the-art remote sensing technologies for PA can be classified as imaging-based and wireless sensing-based approaches. Remote sensing using imaging technologies is based on the application of multispectral cameras to collect images of the field to assess soil moisture and crop stress using airborne instruments^21^. However, multispectral imaging techniques are limited to the analyses of topsoil and cannot be used for subsoil moisture measurement. In addition, the crop stress analysis is not fully reflective of the VWC in the root zone due to the time delay in the water uptake and the complexities in correlating the non-uniform water transport exhibited by plants to the root water stress^22^. The lack of access to subsoil conditions limits the applications of imaging-based technologies to topsoil and plant health monitoring.

As an alternative to imaging-based technologies, wireless sensing using the Internet of Things (IoT) has emerged as a smart farming solution for real-time monitoring of subsoil and root zone^23^. Most IoT networks merge existing wireless communication standards with an array of active electronic sensors in the field. While IoT for PA has been successful at improving resource management and food productivity, it comes with certain pitfalls. Most IoT sensors carry onboard batteries (active) and electronic chips (chipped)^24^, which increase the cost of manufacturing and assembly, and therefore impose a limit on the number of nodes that can be deployed in the field^25^. Furthermore, chipped sensors are not environmentally friendly as the leakage of harmful chemicals, such as lithium and lead, from batteries^26^ and ICs^27^ can pollute the soil as well as water bodies after their functional obsolescence following the crop season.

To address the drawbacks of chipped sensors, chipless wireless sensors have been widely used as a sensing solution as they do not require electronic chips or batteries to function^28^. The low-cost implementation capabilities enabled by additive manufacturing (AM) methods and high-throughput process steps that do not require component assembly have made chipless sensors a common choice for inexpensive humidity sensing^29^, gas detection^30^, and structural health monitoring^31^. While chipless sensors circumvent a lot of the problems posed by alternate sensor technologies used for PA, they have not been used for soil health monitoring due to certain challenges that are crucial for PA. The main limitation of the reported chipless sensors is that the dimensions of the sensors make their deployment cumbersome, since large-sized sensors are unsuitable for automated distribution in the fields. Secondly, existing chipless sensors are mostly made of non-biodegradable metals and polymers, such as copper and Flame Retardant glass-reinforced epoxy resin laminate (FR4), which can lead to the degradation of soil quality. Thirdly, while most of the chipless sensors are tested in the lab, their dependability in field conditions is often not reported. This requires the development of a portable system suitable for testing the sensors in agricultural fields. Finally, it is critical to understand the lifetime and degradation behavior of biodegradable sensors in order to estimate their reliable functional period in the field^32^.

To overcome the challenges of miniaturization, biodegradability, portability, and reliability identified in the existing PA sensor network development, here, we demonstrate a Degradable Intelligent Radio Transmitting Sensor (DIRTS). In this study, electrically small antenna (ESA) technology was used in conjunction with additive manufacturing techniques to develop DIRTS in order to address the requirements of biodegradability and miniaturization of sensors. A systematic study of ESAs was performed to identify the optimum size requirements for a sensor that operates in the frequency range that is ideal for soil monitoring in all moisture conditions. Subsequently, biodegradable and RF (Radio Frequency)-compatible materials were identified to design and manufacture eco-friendly sensors. After material identification, a scalable additive manufacturing technique was employed for fabricating DIRTS using 3D printing of biodegradable substrates and laser processing of adhesive-backed biodegradable metallic sheets. To demonstrate the working of the sensors in both lab and field conditions, a lightweight, portable readout system was developed and integrated into a drone for real-time measurements. Since aerial vehicles have a widespread application in PA, drone-based measurements of DIRTS were performed in an agricultural field to illustrate the real-life applicability of the sensors as well as the potential integration of the portable system into agricultural drone technology. Finally, a systematic approach was elucidated to study the degradation rate of the sensors in the soil in order to estimate the lifetime and decomposition time of the sensors in the field.

## Results and discussion

### System configuration

**Figure 1:**
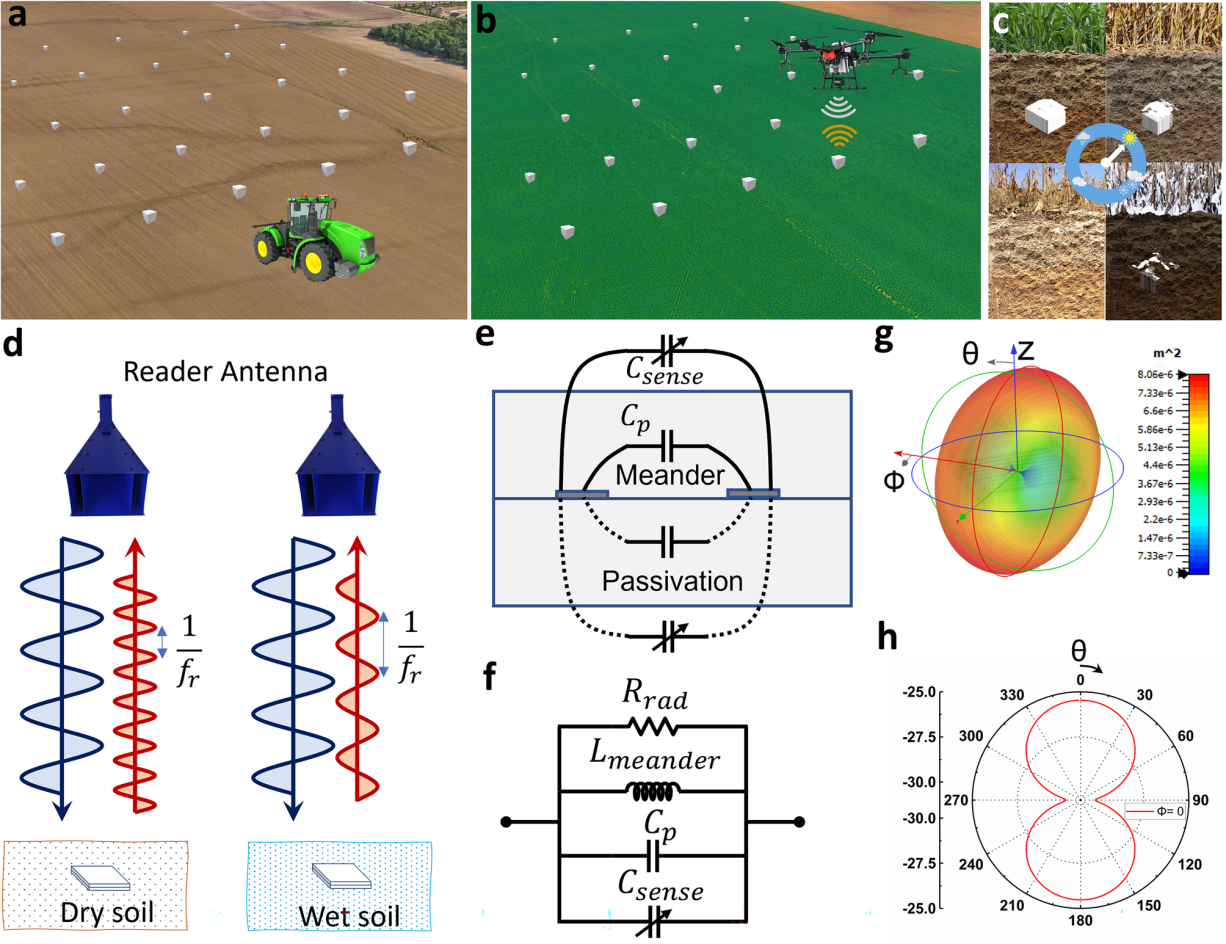
Conceptual illustration of the timeline and working of the sensor tags (**a**) Sensor tags are distributed in the fields using a sower machine or an automated dispenser at the onset of the crop season. (**b**) A drone carrying an RF reader reads each of the sensor tags in the network during the crop season. (**c**) After the crop season, the sensor tags undergo a gradual process of biodegradation through the following seasons. (**d**) System configuration with the reader antenna interrogating the sensor tags buried in the soil. Maximum backscattering is obtained at the resonant frequency, ${f}_{r}$, and ${f}_{r}$ varies based on the dryness or wetness of the soil. (**e**) Cross-sectional illustration of the capacitance distribution in a meander line structure encapsulated with passivation layers that forms DIRTS. (**f**) Equivalent circuit diagram of DIRTS (**g**) 3D far-field pattern of DIRTS demonstrating the maximum direction of radiation along the z-axis (**h**) Cross-sectional schematic at $\Phi$=0 demonstrating the orientation dependence and the main lobe direction of DIRTS.

The chipless sensor tags were designed to endure and operate through the agricultural cycle that comprises seed sowing, crop growth, fertilizing, and harvesting. At the onset of the crop season, furrows of suitable depths are created to distribute the sensor tags alongside the seeds with the help of a seed planter such that each seed has a sensor tag in its vicinity to monitor the soil health parameters surrounding the seed (Fig. 1a). Once a batch of sensor tags is buried, a drone carrying a reader module interrogates the sensors tags by frequently scanning the field to collect information on soil properties (Fig. 1b). While scanning the field, an interrogation signal is sent by the reader module on the drone targeting the sensor tags and the reflected signal from the sensor tag is collected. The resonant peak on the reflected signal spectrum is dependent on the soil conditions and can be correlated to specific soil parameters, such as VWC. The collected information as well as the location of the sensor tag can be transmitted to the receiving station using long-range transmission gateways. Since each measurement takes less than a minute, the drone can cover a large area of land in a few hours. To reduce the environmental impact of subsoil sensors, the sensor tags are made of biodegradable materials that allow a few months to years of operation without performance depreciation followed by a slow controlled degradation. The rate of degradation depends on various factors, such as the moisture content and the microbial activity in the soil, which fluctuate based on weather patterns and seasons (Fig. 1c). DIRTS, being a biodegradable chipless sensor tag, is designed and manufactured to meet these two requirements—perform reliably through the crop season and disintegrate into environmentally friendly products after its lifespan.

The Schematic in Fig. 1d illustrates the working principle of the system. A chipless sensor tag consists of a resonant structure capable of backscattering the incident signal while embedding an electromagnetic signature in the form of resonance in the reflected signal. When the reader antenna sends an interrogation signal to the sensor tag, the sensor tag backscatters signals with maximum amplitude at its resonant frequency. The resonant frequency of a chipless sensor tag depends broadly on two parameters—the geometry of the metallic pattern that forms the resonator and the effective dielectric constant of the medium in the vicinity of the resonator. For a sensor tag made of microstrip lines, the resonant frequency is given by the equation^33^,

1 $$f_{r} = \frac{c}{{2L_{r} \sqrt {\varepsilon_{eff} } }}$$

Where ${f}_{r}$ stands for the resonant frequency; $c$, the speed of light; ${L}_{r}$, the length of the resonator; and ${\varepsilon }_{eff}$ the effective dielectric constant of the media surrounding the sensor tag. For our application, the sensor tag is buried in the soil the dielectric constant of which changes based on the VWC of the soil. Since ${\varepsilon }_{eff}$ when the soil is dry is lower than ${\varepsilon }_{eff}$ when the soil is wet, ${f}_{r}$ of the sensor tag is higher in dry soil than in wet soil.

To interrogate the sensor tag from a drone, a custom-designed readout unit that consists of a low-payload, lightweight, portable reader antenna was developed and integrated into the drone. The portable reader antenna on the drone operates in a dual-polarization mode in conjunction with a depolarizing sensor tag to reduce the noise margin of the ${S}_{21}$ spectrum and improve the readability of the backscattered signal (Supplementary Text ST1). To design a miniaturized device, a meander line structure was chosen as a depolarizing sensor tag as it can reduce the size of the device through a folding technique. The cross-sectional schematic of a meander line structure is shown in Fig. 1e. The capacitance distribution associated with the meander line structure in Fig. 1e demonstrates how the structure can be used in a sensing application. The capacitance across the adjacent meander lines forms a fixed distributed capacitance through the passivation layer and is denoted as ${C}_{p}$. Additionally, the electric field lines traverse through the medium surrounding the sensor tag and form a distributed variable capacitance denoted as ${C}_{sense}$, the value of which changes based on the dielectric properties of the medium. The electrical equivalent of the sensor tag is shown in Fig. 1f. The inductance (${L}_{meander}$) of the meander line structure depends on the length and width of the traces. The inductance, ${L}_{meander}$, and the overall capacitance, ${C}_{p}+{C}_{sense}$, form a tank circuit that resonates at ${f}_{r}$. Since ${C}_{sense}$ is the only variable in the circuit, a correlation can be obtained between ${C}_{sense}$ and ${f}_{r}$, and hence between ${\varepsilon }_{eff}$ and ${f}_{r}$. The radiation resistance (${R}_{rad}$) of the structure depends on the geometry of the structure as well as the wavelength of operation. The effect of radiation resistance can be illustrated by simulating a meander line structure in CST microwave studio. Figure 1g shows the far-field radiation pattern of a meander structure with 10 vertical segments. Meander line structures provide a donut-shaped radiation pattern with maximum radiation along the line of sight of the center of the structure and minimum radiation in the lateral direction. Figure 1h shows the scattering pattern when $\phi$ =0°. The scattering pattern indicates that the main lobe direction is at $\theta$ = 0°, which is along the line of sight of the center of the structure. The radiation pattern obtained from the simulation results shows the possibility of reading the sensor tags using a drone scanning over the field at a reasonable elevation with very high directionality in the angular range of $\theta$ = 0° to 45° Although the radiation intensity of the meander line structure is orientation-dependent, the high directionality of the structure between 0° and 45° helps in reducing cross talk and interference while measuring multiple sensor tags using the drone.

### DIRTS design

**Figure 2:**
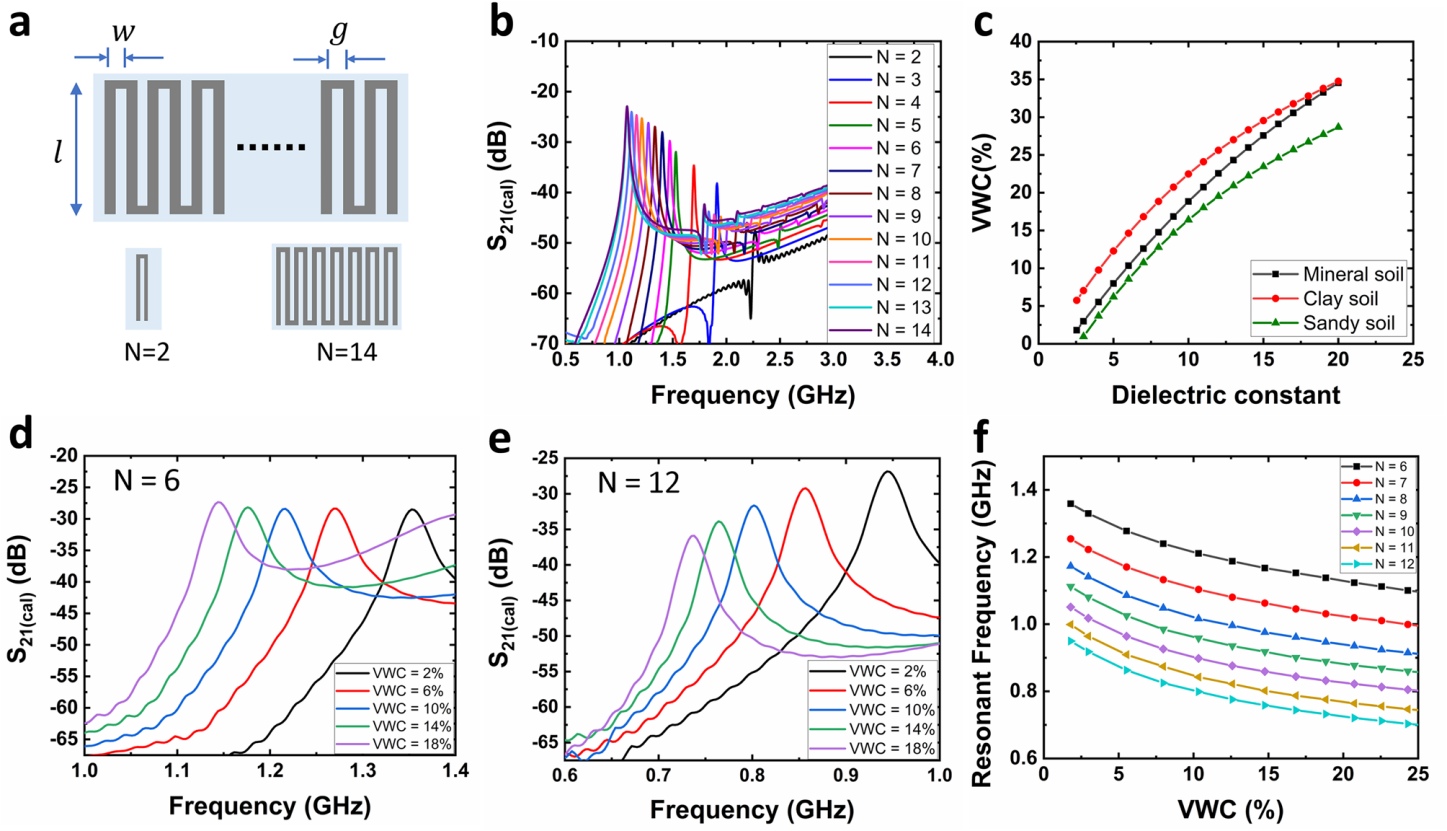
Schematic illustration of meander line structure and simulation-based optimization. (**a**) Meander line structure formation starting from N = 2 to N = 14 with optimization variables indicating various dimensions. (**b**) Simulation results showing ${S}_{21(cal)}$ vs. frequency plots for N ranging from 2 to 14. (**c**) Demonstration of empirical equations demonstrating ${\varepsilon }_{eff}$ as a function of VWC for mineral soil, clay soil, and sandy soil. (**d**) Simulation results for N = 6 showing ${S}_{21}$ vs. frequency plots for various VWC values. (**e**) Simulation results for N = 12 showing ${S}_{21}$ vs. frequency plots for various VWC values. (**f**) Extracted values of ${f}_{r}$ obtained from simulations plotted as a function of VWC for N in the range 6 to 12.

To identify the optimum geometry and length of the sensor tag required to achieve an effective performance within the frequency range of interest, meander line antennas of various lengths were investigated. Meander lines are formed by folding a microstrip line^34^ into a specific number of vertical segments (*N*) of length, $l$, spaced apart by a gap, $g$, and shorted on alternating ends (Fig. 2a). A range of values of N was analyzed in order to make the sensor tag effectively small and obtain a frequency of operation that offers the maximum depth of penetration of RF signals. The size of the sensor tag was limited to 2 cm × 2 cm, a typical dimension of sensors that can be distributed easily and automatically in the field using a seed planter. From a practical manufacturing point of view, the width of the structure ($w$) and the gap between two vertical segments ($g$) were fixed at 1 mm. Finally, the upper limit of the frequency of operation was decided based on the depth of penetration and was identified as 1.5 GHz^35^.

To meet these restrictions, various values of $N$ ranging from 2 to 12 were simulated (Supplementary Text ST2). The simulation results in Fig. 2b show that the minimum value of $N$ required to obtain ${f}_{r}$ ≤1.5 GHz is 6. While increasing $N$ from 6 provides a lower ${f}_{r}$ and as a result, a higher penetration depth, increasing $N$ beyond 12 does not yield any significant variation in ${f}_{r}$ . After identifying the range of $N$ as 6–12, sensor tags with various values of N between 6 and 12 were investigated to identify the design that provides the maximum sensitivity to changes in the VWC of the soil surrounding the sensor tag. For simulating the VWC of the soil that varies with the dielectric constant of the soil, the Topp equation ^36^ was used, as it provides a correlation between VWC and ${\varepsilon }_{eff}$ for soil found in agricultural fields (Fig. 2c, Supplementary Text ST3). Topp equation can be written as,

2 $$VWC=4.3\times {10}^{-6}{\varepsilon }_{eff}^{3}-5.5\times {10}^{-4}{\varepsilon }_{eff}^{2}+2.92\times {10}^{-2}{\varepsilon }_{eff}-5.3\times {10}^{-2}$$

Simulations were performed to study the effect of VWC on ${f}_{r}$ for $N$ from 6 to 12 with the help of the Topp equation. As shown in Fig. 2d, the sensor tag with N = 6 demonstrated an average change of 16.29% when VWC was changed from 2 to 18%. For the same change in VWC, N = 12 demonstrated an increased frequency shift of 21% (Fig. 2e). As shown in Fig. 2f, among various values of N ranging from 6 to 12, N = 10 provides the optimum frequency range as it operates at a center frequency of ~ 915 MHz, which is the center frequency of the Industrial, Scientific, Medical (ISM) band widely used for commercial applications. As a meandered line structure with 10 vertical segments provides a 25-fold area reduction when compared with a microstrip line of length 10 cm^33^ that satisfies the same frequency range, it constitutes an ideal miniaturized sensor for ISM band applications.

After optimizing the structure of the sensor tag, simulations were performed to estimate the radius of the sensitivity zone of the sensor tag (Supplementary Text ST4 and Fig. S1). Simulations revealed that DIRTS is sensitive to changes in VWC within 1 cm of its proximity. A localized sensitivity zone of 1 cm allows a wide-area distribution of sensors without interference or coupling between them, which is critical for PA as the recommended sampling distance for large-scale distribution of sensors is 30 m^37^. As DIRTS can provide a high-resolution mapping of soil moisture across the field with the help of its localized sensitivity zone, the spatial variability of VWC in the soil can be accurately captured.

### Sensor fabrication

**Figure 3:**
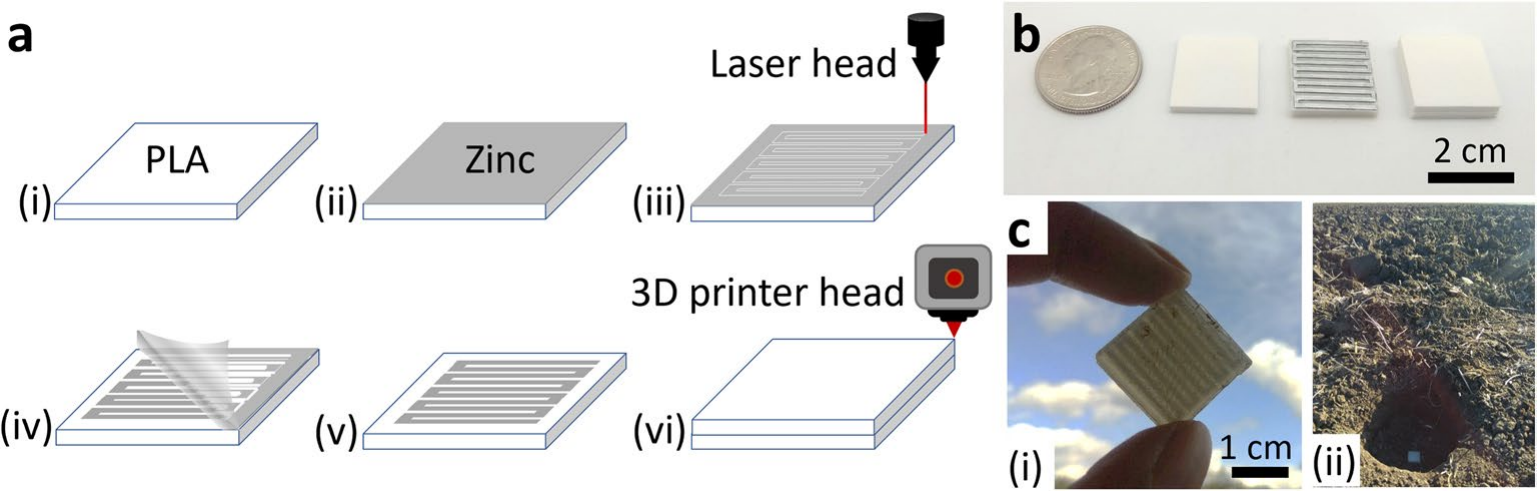
(**a**) Conceptual illustration of the fabrication of DIRTS (i) 3D printed PLA substrate (ii) Zinc tape attached to the top of the PLA substrate (iii) Laser cutting the zinc layer to engrave a meander line pattern (iv) Removal of excessive zinc tape from the surface (v) Meander line structure on the PLA substrate after the removal of the remnant zinc tape (vi) 3D printing the PLA superstrate to passivate the sensor tag. (**b**) Images of DIRTS in its various stages of fabrication. (**c**)Images of DIRTS that demonstrate (i) significant size reduction and (ii) portability for field applications.

The structure of the sensor comprises a conductive metal trace encapsulated between two layers of biodegradable polymeric material. To achieve scalable manufacturing of the sensors, 3D printing^38,39^ and laser processing^40,41^ have been widely employed in previous studies. As shown in Fig. 3, in this process, the conductive traces were laser cut and were encapsulated with 3D printed biodegradable polymers. Since the most commonly used material combinations for chipless sensors, such as copper on FR4^42^ and aluminum on PET/paper^43^, are not biodegradable, zinc on polylactic acid (PLA) is used as the biodegradable alternative. Zinc has a conductivity of the order of ~ 10^7^ S/m and has a higher Q-factor than other biodegradable metals, such as Iron, at high frequencies^44^. Moreover, the manufacturing process of zinc is easier due to the availability of metalized tape that can be patterned with laser processes^45^. PLA formed the substrate and superstate and was manufactured with the help of 3D printing techniques (see *Methods* for details). PLA is a commercially available thermoplastic, widely used in organic electronic devices^46^, wireless drug delivery systems^47^, and printed circuit boards^48^, due to its low melting temperature, low cost, biodegradability, and moisture resistance. As a result, PLA can form a moisture-resilient coating around the biodegradable zinc traces, thereby preventing the degradation of the conductive properties of zinc. PLA’s unique characteristics allow them to be a suitable structural material for DIRTS that is required to maintain stable operation for a certain duration inside the field but gradually degrade over a long period of time.

### Portable DIRTS reader

**Figure 4:**
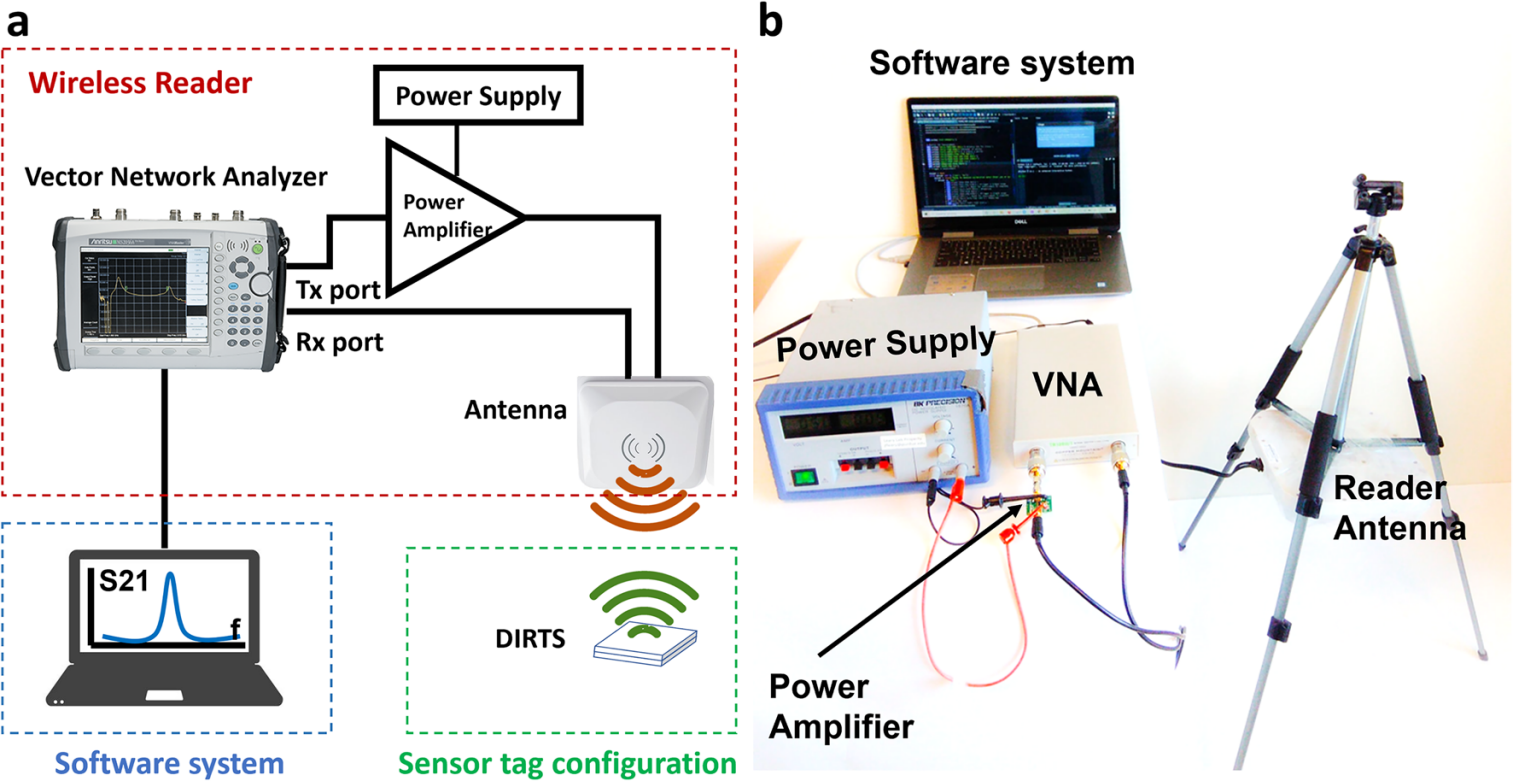
Wireless reader development (**a**) Schematic illustration of the various building blocks of the custom-designed portable readout unit. (**b**) Picture of the implemented portable system consisting of the antenna, VNA and its accessories, and the software system.

To integrate the reader onto a drone and perform wireless measurements of the fabricated device, the readout system was miniaturized into a mountable low-payload unit. The custom-designed lightweight unit consisted of a portable Vector Network Analyzer (VNA) connected to a portable dual-polarized antenna through a power amplifier (Fig. 4a). The output power from the transmission port of the VNA was amplified by the power amplifier and was radiated from the vertically polarized ridge of the portable antenna. The horizontally polarized ridge of the antenna was connected to the receiver port of the VNA to collect and analyze the backscattered signals from the sensor tag. The VNA communicated with a customized software system automated with a python interface to facilitate the on-demand availability of VWC information to the field station.

The fully assembled version of the custom-designed miniaturized reader unit that consists of a portable VNA, power amplifier, and a lightweight antenna is demonstrated in Fig. 4b. The total power delivered to the antenna’s vertically polarized ridge was 12 dBm. The antenna connected to the output of the power amplifier was a pair of cross-polarized log-periodic antennas that could provide a gain of 9 dBi in the 698–960 MHz band covered by DIRTS as shown in the simulations and was, therefore, ideal for our measurements.

### Experimental studies

**Figure 5:**
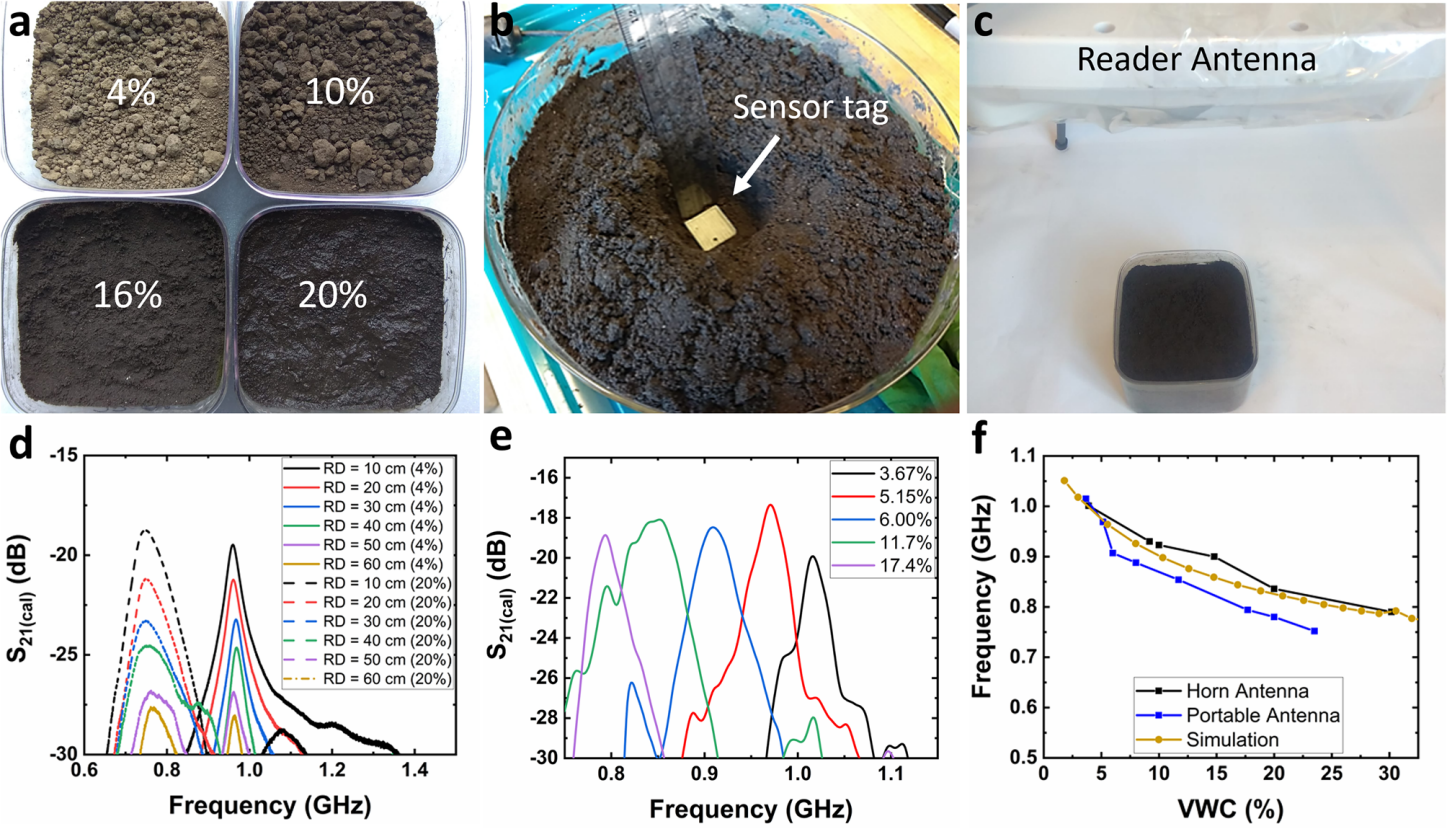
Experimental studies with DIRTS buried in the soil samples (**a**) Soil samples with VWC 4%, 10%, 16%, and 20%. (**b**) Demonstration of the placement of DIRTS at a depth of 5 cm in a soil sample. (**c**) The portable antenna aligned centering the sample to obtain maximum reflections. (**d**) ${S}_{21(cal)}$ vs. frequency measured with the portable reader for different read distances (RD) and VWCs demonstrating a reduction in ${S}_{21\left(cal\right)}$ as a function of RD (**e**) ${S}_{21(cal)}$ vs. frequency measured with the portable reader for different VWCs when RD = 40 cm showing a shift in ${f}_{r}$ with VWC. (**f**) Resonant peaks extracted from ${S}_{21(cal)}$ vs. frequency curves measured with the portable reader plotted as a function of VWC. Measured results from the anechoic chamber experiments as well as the results from simulations plotted for comparison.

To test the performance of the sensor tags in a wide range of relevant field conditions, soil samples with different volumetric water contents were prepared, as shown in Fig. 5a. For the experiments, the sensor tag was placed at a depth of 5 cm in the soil sample (Fig. 5b) as the optimum seeding depth for small grains is 4–5 cm. The soil sample with the sensor tag buried underneath was placed in the line of sight of the reader to obtain maximum reflections (Fig. 5c). The sensor performance was tested in lab conditions using a portable antenna and was compared with the readings from a stationary horn antenna to assess the effectiveness of the portable readout system. The sensor performance was obtained from the resonance spectrum illustrated in the form of an ${S}_{21}$ vs. frequency plot, where ${S}_{21}$ is the ratio of the backscattered power received by the reader to the power transmitted by the reader. The ${S}_{21}$ measurements were calibrated in order to eliminate the environmental noise and ${S}_{21(cal)}$ was reported in the results (See *Methods* for calibration).

To estimate the maximum read distance (RD) of DIRTS, a dry soil sample of VWC = 4% and a wet soil sample of VWC = 20% were tested by varying the elevation of the portable reader from 10 to 60 cm (Fig. 5d). In both soil conditions, ${f}_{r}$ remained unchanged as ${f}_{r}$ was not a function of RD, as shown in Eq. (1). However, in both cases, the amplitude of the resonant peak reduced by ~ 9 dB as RD was varied from 10 to 60 cm. Considering the potential losses from the misalignment with respect to the reader and obstacles in the path, a safe noise margin for the read distance was defined at − 25 dB. In both cases, the amplitude of the resonant peak surpassed the noise margin when RD > 40 cm. Since the backscattered signal substantially weakened below the noise margin, 40 cm was defined as the maximum read range of the sensor tag when buried in the soil at a depth of 5 cm. Since the critical measurement period for most cereal grain crops is the initial few weeks that consist of seed germination and seedling growth, the interference caused by small seedlings will be negligible at a read distance of 40 cm. In addition to the read distance, the sensitivity of the sensor tag to angular orientation was tested by varying $\theta$ when placed at RD = 40 cm (Supplementary Text ST5). The results indicated that the optimum angular orientation required to obtain an amplitude reduction of < 3 dB is 0° to 45° (Fig. S2) corroborating the simulation results obtained from the radiation pattern in Fig. 1h.

After identifying the optimum read distance as 40 cm and the best angular orientation as 0°–45° the response of the sensor tag to varying moisture conditions was studied using the custom-designed portable reader setup in Fig. 5c. The ground truth VWC measurements were obtained from a Teros 12 reader and were correlated to the ${f}_{r}$ obtained from the portable reader measurements to develop a calibrated curve. As shown in Fig. 5e, when the VWC of the soil was increased from 3.67% to 17.7%, ${f}_{r}$ was reduced from 1.015 GHz to 0.794 GHz, indicating an overall shift of 21.77%. A discernible frequency spectrum was obtained up to 23.5% of VWC. The resonant peaks obtained from the soil experiments were extracted and plotted in Fig. 5f to analyze the results from the simulations, anechoic chamber test, and portable antenna test. As shown in Fig. 5f, the resonance peaks obtained for various values of VWC from the anechoic chamber demonstrate a close match with the readings from the simulations. Figure 5f also shows the results from the portable reader, which illustrate the same trend in the frequency roll-off characteristics as the simulations and provide a reasonable match with the simulations.

To analyze the sensitivity of DIRTS in the soil, the readings obtained from the simulations, horn antenna, and portable antenna were compared using Fig. 5f. In all the cases, DIRTS demonstrated a high sensitivity of above 40% to VWC values below 6%. When the VWC was above 6%, a low sensitivity region was attained where a sensitivity of 9.21 MHz/%, 8.7 MHz/%, and 9.07 MHz/% was observed from the simulations, stationary horn antenna readings, and portable antenna readings, respectively. This study confirmed that the sensor tag provided comparable performances in the presence of a portable reader unit as well as a stationary horn antenna. Furthermore, the simulation results were able to estimate the sensitivity of the sensor tags in the soil with reasonable accuracy. Although the horn antenna could provide readings up to 30% of VWC due to its superior cross isolation, they were limited to laboratory conditions due to their bulkiness and unwieldiness. The portable antenna, on the other hand, could cover the typical VWC readings observed in the agricultural fields while providing easier integration to a low-payload drone and was, therefore, the best choice for drone-assisted measurements in field conditions.

### Biodegradation studies

**Figure 6:**
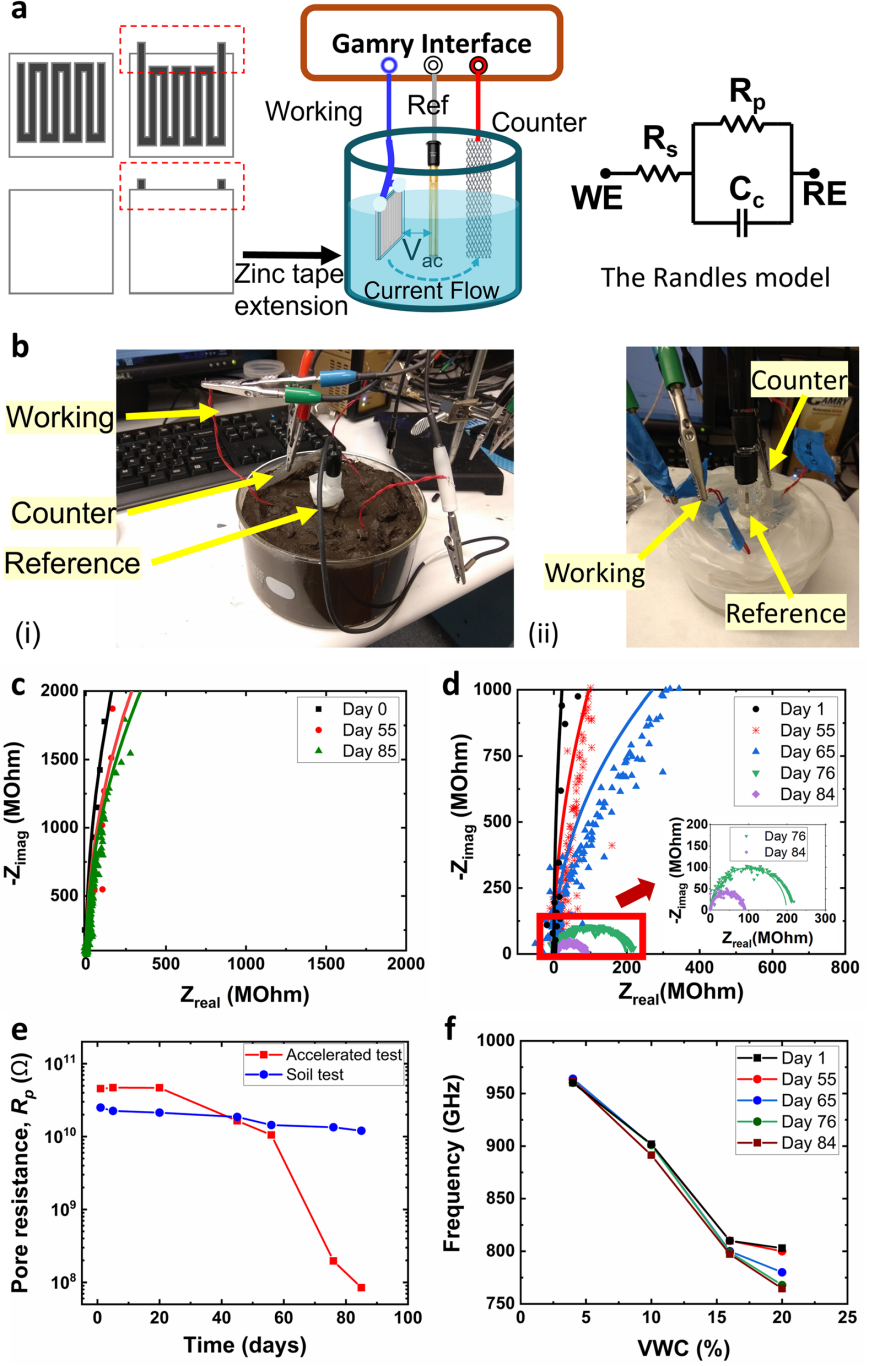
Experimental studies on biodegradation of DIRTS in an accelerated degradation environment and a soil environment (**a**) Conceptual illustration of the sensor tags modified for EIS measurements, the 3-electrode configuration for EIS measurements, and the Randles model used for impedance fitting. (**b**) Images of the experimental setups for (i) the soil test and (ii) accelerated test (**c**) Nyquist plots measured on various days when the sensor tags were in the soil. (**d**) Nyquist plots measured on various days when the sensor tags were in the accelerated degradation environment. The inset features the semicircle patterns on the Nyquist plots. (**e**) Extracted values of ${R}_{p}$ as a function of time for the soil test and the accelerated test. (**f**) Resonant peaks extracted from concurrent RF measurements plotted as a function of VWC.

To assess the overall lifetime of the sensor tags, an accelerated test scenario was created in the lab with protease enzymes that were often found in agricultural fields. The experimental samples were exposed to high levels of protease enzymes to accelerate the process of degradation, whereas the control samples were exposed to the soil for controlled degradation. The enzyme causes the hydrolytic degradation of the PLA encapsulation through a bulk erosion mechanism which is similar to the process of microbial degradation of PLA in soil. The degradation behavior of PLA was examined with electrochemical impedance spectroscopy (EIS) (See *Method*s for details) and the rate of degradation was correlated to the loss of sensitivity of resonant frequency to VWC using wireless measurements. The meander line structure of the sensor tag was connected to the working probe through a simple modification (Fig. 6a). As the porosity of the samples measured by EIS is a reflection of the overall degradation of PLA, a simple Randles model was used to capture the pore resistance, ${R}_{p}$. By measuring ${R}_{p}$ using this real-time analysis method, the rates of degradation of the PLA were obtained from the soil degradation environment (Fig. 6b(i)) and accelerated degradation environment (Fig. 6b(ii)) and were subsequently applied in the calculation of an acceleration factor.

EIS measurements were analyzed with the help of Nyquist plots which provided a vectorial representation of the measured impedance. The Nyquist plots obtained from the soil (Fig. 6c) illustrate a straight line with a slant at low frequencies, which increases from day 1 through day 85 suggesting a relatively small change in the porosity of PLA in a slow degrading environment. In contrast, the Nyquist plot obtained from the accelerated degradation environment in the same time period (Fig. 6d) illustrates a straight line initially, indicating an ideal insulating coating around the sensor tag. Subsequently, the plot transitioned into a semi-circle indicative of the formation of pores and gradual uptake of water, exemplifying the various stages of hydrolytic degradation often observed in polymers. Concurrently, to investigate the effect of degradation on the RF characteristics of the sensor tag, the sensor tags were taken out of the enzyme solution when a significant variation was observed in the Nyquist plots, and wireless sensitivity tests were performed for VWCs ranging from 4 to 20%. To identify the occurrence of a significant event in the degradation behavior, the variation in ${R}_{p}$ was tracked over time. In the initial days of the test, PLA acted as a protective barrier to the enzyme solution, leading to a high ${R}_{p}$ of the order of 10 s of GΩ in both environments (Fig. 6e). However, in the accelerated test, a gradual degradation occurred in the polymer over time, leading to more pores and water uptake into the polymer matrix, which was evident after 55 days of immersion. Although a linear reduction in ${R}_{p}$ was observed from day 55 to day 76 in the soil test, a sharp decrease of 2 orders of magnitude in ${R}_{p}$ was observed in the accelerated test, suggesting a substantial increase in the porosity of PLA. As a corollary, this decline in ${R}_{p}$ was reflected in the wireless sensitivity measurements performed in tandem, where a considerable decrease in ${f}_{r}$ was observed between day 55 and day 76, leading to a 4% loss in sensitivity at 20% VWC (Fig. 6f). The loss of sensitivity can be attributed to the spread of pore formation in the PLA coating causing a rise in the seepage of water from the soil into the pores at high VWC. Overall, the trend in sensitivity deviation aligned with the gradual degradation in the initial phase and a subsequent increase in porosity and the uptake of water into the polymer observed in this study. Similar findings by Hakkarainen et al.^49^ and Maharana T. et al.^50^ , where the hydrolytic degradation of PLA was observed to occur in stages with a slow degradation in the initial stage followed by rapid degradation in the final stage, corroborate the results demonstrated in our study.

To obtain the reliable functional period of DIRTS, an acceleration factor was obtained from the linear region of degradation between day 20 and day 55 as the sensor tags demonstrated a 4% deviation in the RF sensitivity characteristics beyond this range. An acceleration factor of 7.15 was obtained by calculating the ratio of degradation of DIRTS in the accelerated test to that in the soil test in the linear region, indicating that the reliable functional period of the sensor tag in the soil is ~ 1 year with a sensitivity deviation much less than 4%. To estimate the time for complete degradation of PLA, previous studies were analyzed. Karamanlioglu M. et al.^51^ has shown that the biodegradation rate of PLA was approximately 0.02 g/year based on the weight loss method. At the degradation rate reported by Hakkarainen et al.^49^ and Karamanlioglu M. et al.^51^, DIRTS was estimated to undergo complete biodegradation in ~ 80 years. However, in contrast, other commonly used polymers, such as PET and acrylic^52^, have shown a 30-fold lower degradation rate^53^ making PLA the suitable choice for a biodegradable sensor for soil applications.

**Figure 7:**
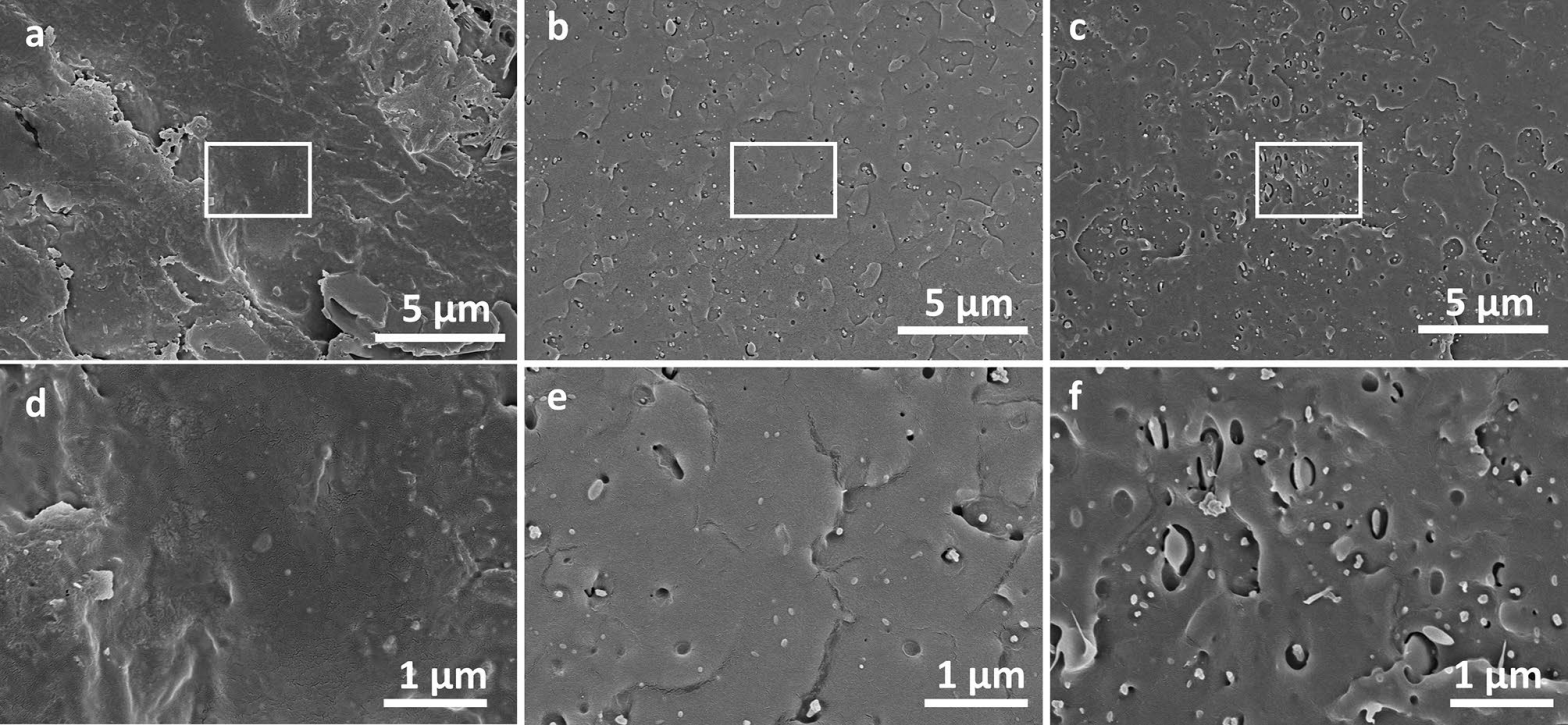
SEM images of DIRTS after having been buried in various media for 3 months (**a**) Cross-section of DIRTS placed in an airtight sealed container demonstrating negligible pore formation after 3 months (**b**) A relatively higher levels of porosity observed in soil (**c**) Maximum porosity observed in accelerated biodegradation environment indicating highest level of degradation. (**d–f**) High magnification images of DIRTS corresponding to the insets demarcated by white boxes in a, b, and c, respectively. [Scale bars: 5 µm for (a-c) and 1 µm for (d-f)].

Finally, to inspect the morphological effects of biodegradation, cross-sectional scanning electron microscopy (SEM) images of DIRTS were taken after 90 days of exposure in a sealed airtight container (Fig. 7a,d), agricultural field (Fig. 7b,e), and enzymatic solution (Fig. 7c,f). Very minimal porosity was observed for samples that were not exposed to enzymatic degradation, whereas the samples placed in the field demonstrated relatively higher levels of porosity. The samples placed in the enzymatic solution, in comparison, demonstrated maximum porosity due to their long-term exposure to an accelerated biodegradation environment (Supplementary Text ST6 and Fig. S3). The difference in ${R}_{p}$ between the samples placed in soil and those in the enzymatic solution was verified with the help of SEM images.

### Field tests with a stationary mount and portable DIRTS reader

**Figure 8:**
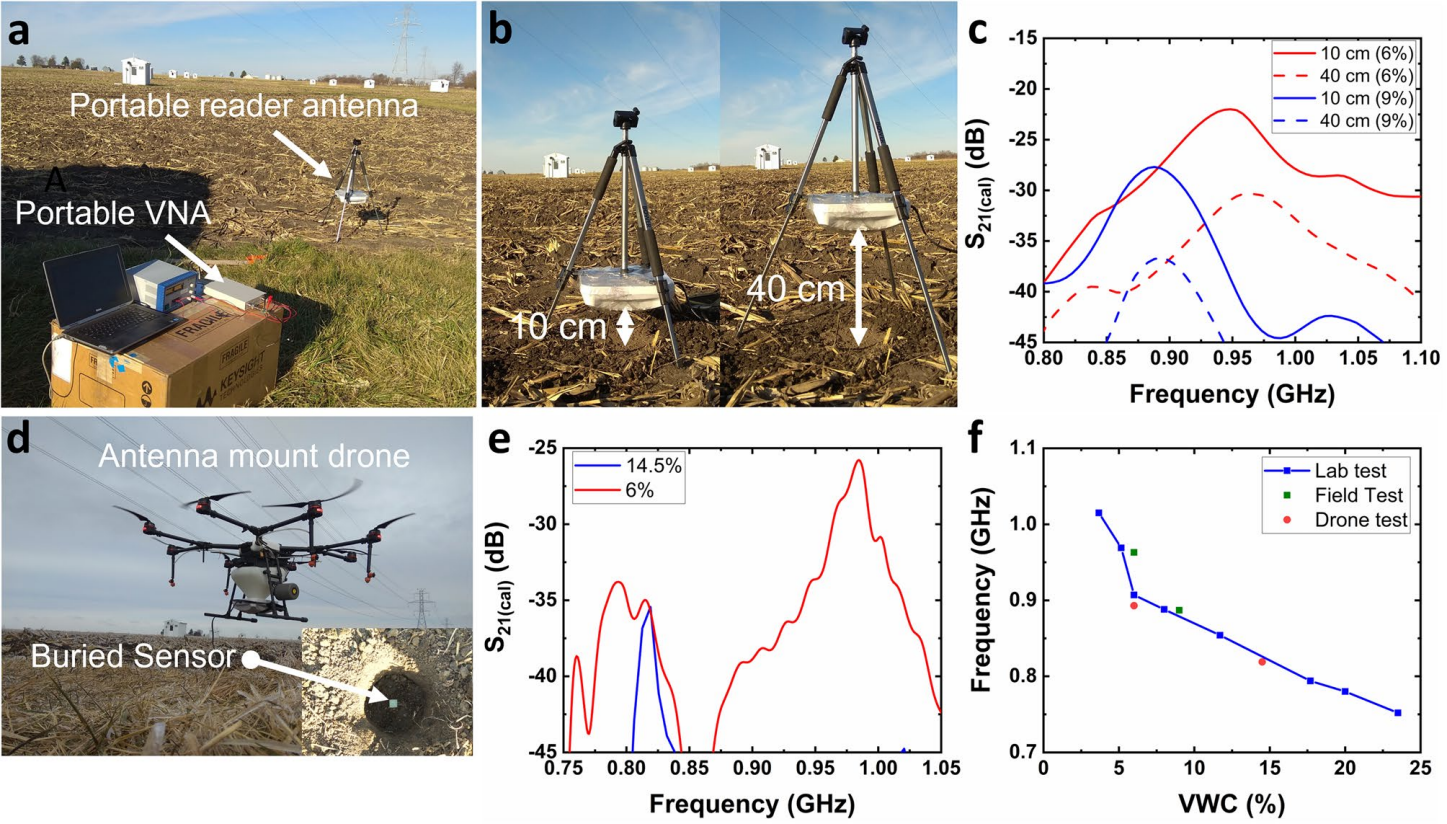
Experimental studies on DIRTS buried in the field when interrogated by a stationary reader and a drone-mounted reader (**a**) Photograph of the portable reader assembly in the field. (**b**) Photographs of the portable antenna loaded onto a stationary mount demonstrating RD = 10 cm, and RD = 40 cm. (**c**) Measured ${S}_{21(cal)}$ plotted as a function of frequency for a VWC of 6% and 9% when RD = 10 cm, and RD = 40 cm. (**d**) Photograph of the drone-mounted antenna hovering over a sensor tag. Inset shows the sensor tag placed at a depth of 5 cm before filling up the bored hole. (**e**) Measured ${S}_{21(cal)}$ plotted as a function of frequency as recorded by the drone from an elevation of ~ 40 cm for ground truth VWCs of 6% and 14.5%. (**f**) Comparison of the results obtained from the portable reader on the stationary mount and the drone with respect to the calibration curve obtained from the lab tests.

Deployments of the portable reader system and the stationary mount for the antenna are shown in Fig. 8a (Supplementary Text ST7). The portable antenna was configured to measure the sensor at two read distances – 10 cm and 40 cm – as shown in Fig. 8b. As shown in Fig. 8c, when RD was increased from 10 to 40 cm, ${S}_{21(max)}$ decreased by 8.33 dB on day 1 and 9 dB on day 2 due to path loss, whereas ${f}_{r}$ demonstrated a negligible deviation with RD, confirming the stability of ${f}_{r}$ to varying elevations of the reader in both VWCs. The values of ${f}_{r}$ extracted from the RF characteristics obtained on two separate days were converted to the corresponding VWCs using the calibration curve developed from the lab test as shown in Fig. 8f. After conversion, DIRTS provided a VWC of 5.2% (${f}_{r}$ = 0.963 GHz) and 8.12% (${f}_{r}$= 0.887 GHz), on day 1 and day 2, respectively. In comparison, the ground truth reader logged 6% on day 1 and 9% on day 2, providing a very low error margin of < 1%, hence, validating the accuracy of the calibration curve for varying field conditions.

After validating the working of the sensors in the field, the efficiency of the drone-mounted portable reader was tested (Supplementary Text ST8). The portable antenna was loaded onto the bottom of the drone by fastening it between the landing gear legs of the drone. The drone demonstrated a highly stable alignment at a height of 40 cm above the ground where the sensor was buried at a depth of 5 cm (Fig. 8d). The post-processed data from the drone measurements is shown in Fig. 8e. In the drone-assisted measurements, DIRTS recorded a VWC of 14.95% (${f}_{r}$ = 0.819 GHz) and 7.3% (${f}_{r}$= 0.984 GHz), on day 1 and day 2, respectively (Fig. 8f). Concurrent ground truth measurements showed a VWC of 14.5% on day 1 and 6% on day 2 demonstrating a good agreement with values obtained from the drone-assisted measurements. Despite the use of a flying drone, a tolerable error margin of < 1.5% was obtained in the high sensitivity region confirming the applicability of DIRTS in real-time measurements.

Finally, as a practical consideration for field measurements, certain environmental parameters that can vary in field conditions, such as temperature, wind, and pressure, were considered. Although soil temperature usually fluctuates between 10 and 40 °C, the dielectric constant of PLA shows negligible sensitivity to temperature in the range of 10–75 °C at GHz frequencies^54^. Since the sensor is buried at a depth of 5 cm, the influence of wind is insignificant. For day-to-day field applications, a sprayer drone can be used for contactless VWC measurements as well as the distribution of pesticides and water, thereby eliminating the soil pressure caused by massive field vehicles. Therefore, by combining chipless wireless sensors with drone-assisted telemetry, DIRTS can operate reliably in field conditions during its functional period in order to measure soil parameters while being resilient to other environmental parameters. As part of future work, DIRTS and multispectral imaging techniques can be used in a complementary configuration, as DIRTS can provide subsoil measurements while multispectral imaging can perform the surface scanning of the field paving new paths in drone-assisted precision agriculture.

## Conclusion

The Degradable Intelligent Radio Transmitting Sensor (DIRTS) reported here enables wireless, in-situ drone-assisted monitoring of the VWC of the soil in various field conditions with high accuracy. DIRTS is a low-cost, fully biodegradable, highly miniaturized sensor that merges the ESA design approach with AM techniques that facilitates laser processing of biodegradable metallic patterns and 3D printing of biodegradable polymers. A CST microwave studio-based simulation platform was used to optimize the geometry of the sensor tags as well as to predict their response to various VWC conditions in the soil. A custom-designed reader unit was developed by integrating a mountable lightweight antenna to a portable VNA to perform measurements in the field. Evaluations of DIRTS were conducted by testing them with a horn antenna in a noise-free anechoic chamber as well as with a custom-designed portable reader unit in a noisy environment and the outcomes validated the simulation results. Initial field studies carried out in an agricultural field with the portable system in the stationary mode and the sensor buried in the soil corroborated the working of the reader as well as the sensors. A proof-of-concept study done by mounting the portable reader antenna onto a sprayer drone and reading the sensors buried in the soil to extract the VWC of the soil accomplished a practical demonstration of DIRTS for drone-assisted PA applications. EIS-based biodegradation study demonstrated the degradation trend of DIRTS in both soil and an enzymatic solution that accelerated bulk erosion. A comparison study of the pore resistance of DIRTS when placed in the soil and the enzymatic solution helped in predicting the percentage of biodegradation in the field years from now using extrapolation techniques. A parallel study on the deviation of sensitivity (VWC vs. ${f}_{r}$) followed by the SEM analysis of the samples supported the findings from the biodegradation study and validated the functional reliability of DIRTS. DIRTS being a device that has demonstrated a rigorous consolidation of miniaturization, additive manufacturing, portability, and biodegradability, we envision its widespread utility in improving agricultural management given the exceptional demand for low-cost, environmentally friendly sensors for PA. The DIRTS technology is transferrable to other applications, such as food packaging, and human health monitoring, where small-sized biodegradable sensors are critically important.

## Materials and methods

### Fabrication of biodegradable sensor tag

The process steps started with 3D printing PLA sheets using an Ultimaker 3D printer. The print core used a 0.4 mm nozzle and operated at a resolution of 0.1 mm. The infill density of PLA was set to 20%. The printing temperature and the build plate temperature were set to 200 and 60 °C, respectively. The PLA sheet was designed to be of size 2 cm × 2 cm × 2.5 mm (Fig. 3a(i)). At a 70 mm/sec speed, an array of PLA substrates can be 3D printed in less than an hour. Once the substrates were 3D printed, zinc tape was cut to size 2 cm × 2 cm and was attached to the PLA substrate (Fig. 3a(ii)). A laser engraver was used to define a meander line pattern on the zinc layer (Fig. 3a(iii)). The remaining zinc tape on the keepout layer was removed (Fig. 3a(iv) so as to finish the pattern formation (Fig. 3a(v)). The Ultimaker 3D printer was used to extrude PLA to form a 2.5 mm thick superstrate (Fig. 3a(vi)). The sensor tag after each stage of fabrication—the formation of PLA substrate, patterning of zinc layer, and extrusion of PLA superstrate – is shown in Fig. 3b. The fully fabricated sensor weighed 4 g and was compact, lightweight, and coin-sized (Fig. 3c(i)). The final figure (Fig. 3c(ii)) shows the sensor tag in an agricultural field.

### Wireless reader implementation

The assembled measurement configuration is demonstrated in Fig. 4b. A commercially available portable VNA from Copper Mountain Technologies was used for the measurements. This VNA can transmit signals from 300 kHz to 1.3 GHz with a resolution of 1 Hz. Although the VNA was able to generate up to 3 dBm of power, the Tx port was calibrated to generate -10 dBm as the excess reflected power could overload the VNA’s receiver port. The power amplifier connected to the output of the VNA was an ADL5911 RF/IF gain block from Analog Devices. The ADL5911 gain block is a broadband amplifier that provides a fixed gain of 22 dB in the frequency range 30 MHz to 6 GHz. A total power of 12 dBm was delivered to the antenna’s vertically polarized ridge. An XPOL-2-5G from Poynting was connected to the output of the power amplifier. XPOL-2-5G is a cross-polarized pair of log-periodic antennas that can provide a gain of 9 dBi and a VSWR of < 2 in the 698–960 MHz band.

### Soil sample preparation

The soil samples were collected from the field and were dried in an oven at ~ 80 °C. The samples were ground and sieved to obtain a homogenous mix of dry soil. The dry soil was methodically sprinkled with water and subsequently thoroughly mixed. The sample was capped at the required VWC levels using a commercially available Teros 12 VWC sensor, which served as the ground truth reader.

### Calibration of wireless measurements

Since the resonance spectrum consists of reflections from both the sensor tag as well as the soil, the reflective and absorptive properties of the soil need to be nullified to minimize noise and to extract the backscattering effect of the sensor tag alone. For noise reduction, a calibration step was performed to obtain ${S}_{21(isolation)}$ by placing the same soil sample without the sensor tag buried in it. ${S}_{21(tag)}$ was obtained from the measurements with the sensor tag buried in the soil sample. The calibrated ${S}_{21}$ vs. frequency plot was obtained by subtracting the ${S}_{21}$ readings with the sensor tag in the soil from the ${S}_{21}$ readings without the sensor tag in the soil ( ${S}_{21(cal)}={S}_{21(tag)}-{S}_{21(isolation)}$).

### Anechoic chamber measurements

Using a noise-free anechoic chamber helps in assessing the performance of the sensor in an environment with minimum electromagnetic interference and maximum echo suppression making the comparison between the results from the simulations and the results from the anechoic chamber easier. The tests in the anechoic chamber were performed with an ETS Lindgren 3164-10 quad ridge dual polarized horn antenna connected to a Keysight E5072A VNA.

### Biodegradation evaluations

The zinc layer of the sensor tag was modified in order to obtain a structure compatible with EIS measurements. Either end of the meandered lines was extended (Fig. 6a) and copper wires were soldered onto the extensions to establish a connection to the EIS equipment. Since the meandered lines were to be protected from the enzymatic solution, the contact region was secured using an epoxy coating.

#### EIS measurements

EIS measurements were performed in a three-electrode configuration, which consisted of a working electrode, a reference electrode, and a counter electrode. A small-signal voltage of peak amplitude 100 mV was applied between the working electrode and the reference electrode. The current flows from the working electrode to the counter electrode. Gamry Reference 600 was used to perform the EIS measurements. The passivated extensions from the meandered lines were connected to the working electrode of Gamry Reference 600. A commercial Thermo Scientific 900,200 Orion Sure-Flow Ag/AgCl Half-Cell Electrode was used as the reference electrode, and a steel mesh was used as the counter electrode. The frequency was swept from 0.1 Hz to 10 kHz to obtain an impedance spectrum from which Nyquist plots were extracted.

#### Biodegradation environments

In the accelerated degradation environment, sensor tags were kept in a solution of proteinase K and Tris–HCl buffer at 35 °C^55^. Proteinase K, extracted from the fungus Engyodontium album, was used in the accelerated biodegradation studies of PLA and PLA polymer blends^56^. In the soil degradation environment, sensor tags were kept in soil maintained at 30% VWC.

### Microscopy

Samples were freeze-dried for 24 h to reduce distortion. Subsequently, they were freeze-fractured to obtain cross-sectional samples. Scanning electron microscopy was performed using a Hitachi S-4800 after Au–Pd coating (SPI sputter coater) to reduce charging.

## Supplementary Information

Supplementary Information.

## Data Availability

The data that support the findings of this study are available from the authors on reasonable request.
