# Supplementary material for: A biodegradable chipless sensor for wireless subsoil health monitoring
Source: Sci Rep. 2022 May 14;12:8011. doi: 10.1038/s41598-022-12162-z (PMC9107491; doi:10.1038/s41598-022-12162-z)
Supplement: Supplementary file 1 — Supplementary Information. [file 41598_2022_12162_MOESM1_ESM.docx]

**Supplementary Information for**

**A biodegradable chipless sensor for wireless subsoil health monitoring**

Sarath Gopalakrishnan^a,c^, Jose Waimin^b,c^, Amin Zareei^b,c^, Sotoudeh Sedaghat^b,c^, Nithin Raghunathan^c^, Ali Shakouri^a,c^, and Rahim Rahimi*^a,b,c^

^a^School of Electrical and Computer Engineering, Purdue University, West Lafayette, IN, USA 47907

^b^School of Materials Engineering, Purdue University, West Lafayette, IN, USA 47907

^c^Birck Nanotechnology Center, Purdue University, West Lafayette, IN, USA 47907

***Email:**  [rrahimi@purdue.edu](mailto:rrahimi@purdue.edu)

Supplementary Information Text

**Text ST1. Dual polarized reader and depolarizing sensor tags**

A dual-polarized reader unit requires a depolarizing sensor tag in order to convert the incident vertically polarized signals into horizontally polarized signals. Depolarizing refers to the ability of sensor tags to transform the polarization of the incident signal from vertical polarization to horizontal polarization or vice versa. The biggest advantage of this method is that it improves the noise margin of the backscattered signal. Since the signal strength of the backscattered signal from the sensor tag is of the order of the leakage from the transmitter to the receiver, it is important to assure that the backscattered signal from the sensor tag has a unique polarization which makes it distinguishable from the leaked signals. Since the transmitted signal is vertically polarized, the receiver is configured to read horizontally polarized signals in order to minimize leakage. The depolarizing nature of the sensor tag is enabled by rotating the sensor tag by 45⁰ about an axis perpendicular to its largest surface at which a maximum signal strength can be attained. Among the most common and simple geometries, microstrip lines demonstrate depolarizing properties. However, based on Equation (1), the length of the microstrip line required to obtain a resonant frequency of 1 GHz is 10 cm which considerably increases the size of the sensor tag. One of the most common geometries that have been used to miniaturize microstrip lines when space requirements are a constraint for RFID applications (SR1) is meander lines as they offer the largest size reduction for a given resonant frequency (SR2).

**Text ST2. Methodology of optimizing the structure of DIRTS**

The dimensions of DIRTS were optimized using simulations in CST Microwave studio. The number of vertical segments ($N$) was parameterized using the simulation tool. A plane wave approximation was assigned to simulate the reader antenna. For this simulation, the background was set as air, and $N$ was increased from 2 to 14. As $N$ was increased, the effective length of the resonator proportionately increased leading to an increase in $f_{r}$. However, the meander line structures reached a frequency saturation point as $N$ was further increased, as reported in previous work (SR3). This saturation point is the lower limit of the frequency.

**Text ST3. Models for correlating the volumetric water content to the dielectric constant of soil**

Several models have been used to draw a correlation between VWC and the dielectric constant of the soil. Jacobsen and Schjonning (SR4) reported a calibration equation for heavy clay soil and sandy soil which included the effects of clay percentage and loamy bulk density. Figure 2C shows how Jacobsen and Schjonning estimated the correlation between VWC and $\varepsilon_{eff}$ for heavy clay soil with a loamy bulk density of 1.2 and clay percentage of 75%, and sandy soil with a loamy bulk density of 1.55 and clay percentage of 10%. Although this model captured the various aspects of soil composition, field soil is highly variable from point to point. Topp equation has been extensively used as a universal model for all types of soil and has been shown to be the best calibration equation when all the soil parameters such as the bulk density, porosity, organic matter, and clay percentage are not known. For mineral soil, the Topp equation has been used in commercial probes.

**Text ST4. Sensitivity zone of the sensor tag**

To obtain the radius of the sensitivity zone of the sensor tag, a simulation-based model was developed (Fig. S1a). In the simulation-based model, a sensitivity zone was created between the sensor tag and the soil environment. The sensitivity zone was modelled as a region of PLA that extends by $r_{s}$, varying equally in all directions from the surfaces of the sensor tag. Simulations were done to find the minimum value of $r_{s}$ at which the sensitivity of DIRTS to the VWC of the soil reduced to < 5%, and that value of $r_{s}$ was defined as the radius of the sensitivity zone of the sensor tag. Using boundary conditions, the background dielectric material was set to soil with a parameterized dielectric constant that could vary the VWC of the soil. To estimate the radius of the sensitivity zone,$r_{s}$ was changed from 2 mm to 10 mm while VWC was changed from ~3 to 35 corresponding to a dielectric constant variation from 3 to 20. The simulation results are shown in Fig. S1b. When VWC was increased from 3 to 35, $f_{r}$ was reduced for all values of $r_{s}$. However, the overall shift in $f_{r}$varied from 21.8% to 5.8% when $r_{s}$ increased from 1 mm to 6 mm. As $r_{s}$ was further increased to 10 mm, the overall shift in $f_{r}$reduced to 3.45% surpassing the threshold of 5%. Since the sensitivity of the sensor tag to VWC became insignificant when the overall shift in $f_{r}$ was less than 5%, the radius of the sensitivity zone was identified as 1 cm.

**Text ST5. Angular dependence of the sensor tag**

To investigate the effect of the orientation of the depolarizing sensor tag with respect to a fixed position of the reader, an experiment was conducted. In this experiment, the sensor tag was initially placed facing the reader ($\theta=0⁰$) and was subsequently rotated along $\theta$ while the reader remained fixed (Fig. S2a). Figure S2b demonstrates the variation in the amplitude of $S_{21(cal)}$ as a function of frequency for various values of angular rotation, $\theta$. The angle, $\theta$, at which the radiation intensity reduces by 3 dB in amplitude is the elevation beamwidth of the sensor tag and it signifies the range of orientations of the sensor tag in the vertical plane within which the reception of the signal is best. As shown in Fig. S2b, the amplitude of $S_{21(cal)}$ was reduced only by 1.77 dB when $\theta$ was increased from 0⁰ to 45⁰, but the overall change in the amplitude of $S_{21(cal)}$ surpassed the 3 dB cutoff when $\theta$ was increased to 60⁰. Since the amplitude of the sensor tag became feeble and less detectable when it reduced by more 3 dB, the sensor tag’s optimum angular orientation in the $\theta$-domain was identified as 0⁰ to 45⁰. This results also matches the simulation results of the radiation pattern (Fig. 1h) where the amplitude surpassed the 3 dB cutoff when $\theta$ was increased from 0⁰ to 60⁰. As $\theta$ approached 90⁰, the sensor tag became undetectable as the amplitude sharply reduces to a minimum. However, in a practical scenario, this blind spot can be circumvented by using two sensor tags with complementary orientations or by adjusting the orientation of the reader.

**Text ST6. Porosity analysis of DIRTS**

Cross-sectional SEM images in Fig. 7 display the porosity variation of the DIRTS samples representing different polymer degradation levels upon exposure to soil and enzymatic solution media. The samples placed in soil (Fig. 7 b, e) and enzymatic solution (Fig 7. c, f) demonstrated a significant increase in pore diameters as compared to the fresh samples (Fig 7. a, d). Pore size distribution analysis performed by ImageJ software (Fig. S3) revealed an average pore size estimated about 148 nm, 334 nm, and 600 nm for the fresh samples, samples exposed to soil, and samples placed in an enzymatic solution, respectively. The increased pore size for the samples exposed to the soil could be attributed to the biological activity within the soil that led to polymer degradation. The accelerated polymer degradation in the enzyme solution resulted in a further increase in pore diameters representing higher degradation of the polymer. These results are in confirmation with the calculated $R_{p}$ values for the samples.

**Text ST7. Wireless measurements in the field with a portable reader**

The field tests are conducted at Purdue University’s Agronomy Center for Research and Education, a campus-based field research station. The field tests were done on two different days to verify the working of the sensors in varying conditions. Before testing the readability of the sensor using a drone-mounted portable reader, the sensors needed to be verified in field conditions. Since the field conditions varied from day to day, Teros 12, a commercial VWC reader, was used for ground truth measurements.

**Text ST8. Drone assisted wireless measurements**

For the experiments, Agras MG-1P from DJI was identified as a great candidate since this octocopter was designed specifically for use in agriculture. A remote controller was used to enable liftoff and to control the flight of the drone. Agras MG-1P has a takeoff weight of 23.8 kg and has an 8-rotor propulsion system that provides a highly stable alignment with centimeter-level positioning accuracy. Since the portable antenna weighed only ~ 1.55 kg, the drone could easily carry the portable antenna and hover over the field without considerable spatial misalignment. Moreover, the drone’s A3 remote controller offered a range of up to 3 km with high hovering accuracy and operated in the 2.400-2.483 GHz band without interfering with the portable antenna that worked in the 0.4 -1.3 GHz band. The drone can perform multi-tasking for precision agriculture by integrating the portable reader dedicated for chipless sensing to its preexisting features, such as a spraying system, FOV cameras, and a high-precision obstacle avoidance radar module that operates in the 24.00-24.25 GHz band. The multi-tasking capabilities of agricultural drone technology can reduce the cost and time of PA processes throughout the crop season.

**Supplementary Information – Figures**

**
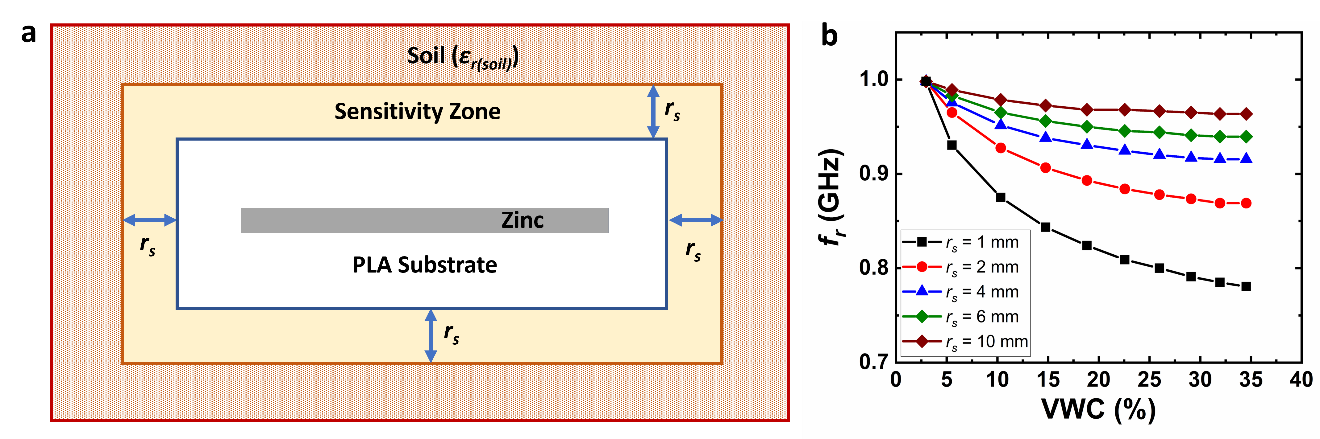
**

Figure S1. Estimation of the radius of the sensitivity zone. (a) Simulation model depicting the sensitivity zone around the sensor tag (b) Simulation results demonstrating the sensitivity variation as a function of $\boldsymbol{r}_{\boldsymbol{s}}$.


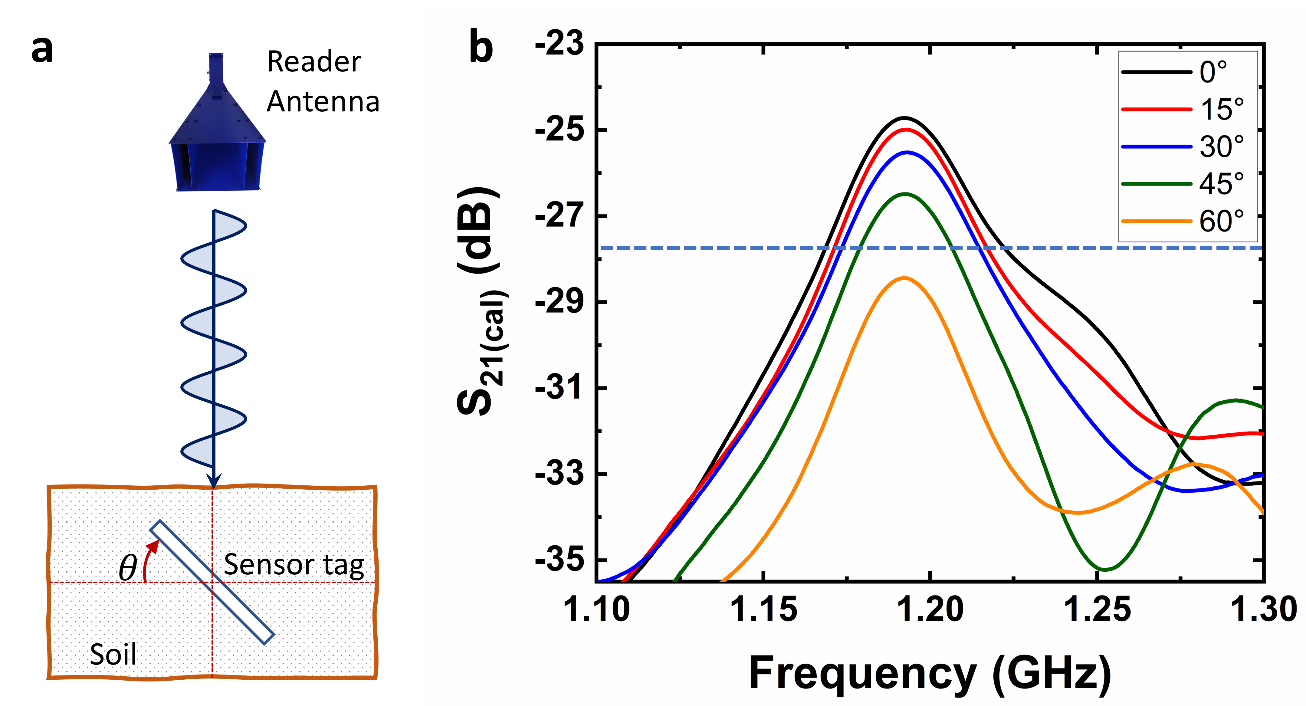


Figure S2. Estimation of orientation dependence of the sensor tag (a) Schematic illustration of the orientation dependance test (b) Experimental results demonstrating the orientation dependence of the sensor tag with $\boldsymbol{\theta}$ changing from 0⁰ to 60⁰ along the vertical plane.


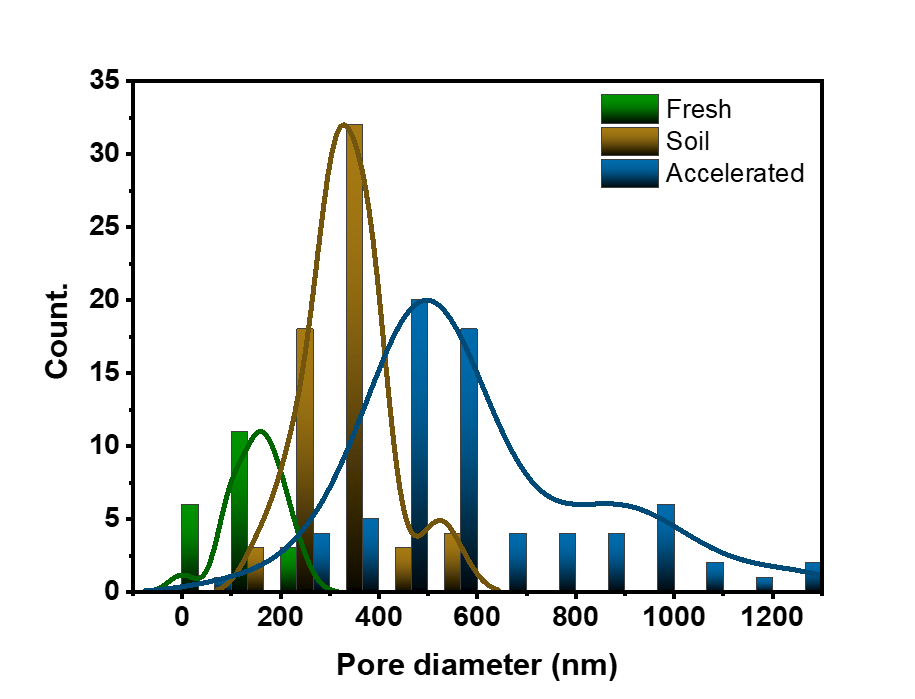


Figure S3. Pore size distribution of the fresh sample, sample buried in soil, and sample in the enzyme solution.

**SI References**

1. G. Marrocco. Gain-optimized self-resonant meander line antennas for RFID applications. *IEEE Antennas and Wireless Propagation Letters* **2**, 302-305 (2003).
2. G. Leon, R.R. Boix & F. Medina. A comparison among different reduced-size resonant microstrip patch. *Microwave and Optical Tech. Letters* **29**, 143-146 (2001).
3. O. O. Olaode, W. D. Palmer & W. T. Joines. Characterization of Meander Dipole Antennas with a Geometry-Based, Frequency-Independent Lumped Element Model. *IEEE Antennas and Wireless Propagation Letters* **11**, 346-349 (2012).
4. Jacobsen, Ole H., & P. Schjonning. Comparison of TDR calibration functions for soil water determination. In *Proceedings of the Symposium: Time-Domain Reflectometry Applications in Soil Science*, 25-33 (1995).
